# Innovative double suturing technique for gastric endoscopic hand suturing after endoscopic submucosal dissection: a case report and technique description

**DOI:** 10.1055/a-2271-7108

**Published:** 2024-03-20

**Authors:** Takuma Okamura, Tomonari Ikeda, Tetsuro Honda, Tatsuki Ichikawa, Kazuhiko Nakao

**Affiliations:** 1 13650 Department of Gastroenterology, Nagasaki Harbor Medical Center, Nagasaki, Japan; 2 200674 Department of Comprehensive Community Care Systems, Nagasaki University Graduate School of Biomedical Sciences, Nagasaki, Japan; 3 Department of Gastroenterology, Honda Internal Medicine and Endoscopy Clinic, Nagasaki, Japan; 4 13650 Department of Gastroenterology, Nagasaki Harbor Medical Center, Nagasaki, Japan; 5 200674 Department of Gastroenterology and Hepatology, Nagasaki University Graduate School of Biomedical Sciences, Nagasaki, Japan

Endoscopic hand-suturing has been reported to achieve a more robust gastrointestinal mucosal closure for mucosal defects following gastric endoscopic submucosal dissection 1 2 . While it is expected to reduce the risk of postoperative bleeding and perforation 3 , reports on its usefulness in the real world are limited because its clinical application is relatively recent, and its effectiveness remains controversial. Closure of only the mucosal layer for mucosal defects often results in a submucosal dead space and dehiscence 4 5 . Therefore, we devised a technique involving double suturing using two threads.

The innovative double suturing method was performed as follows. We used a single-channel scope (GIF-XZ1200; Olympus, Tokyo, Japan) with a cylindrical hood attached, and two types of barbed suture (VLOCK1204, 3/8 CIRCLE 17 mm CV-15 for the first suturing; VLOCK1704, 5/8 CIRCLE 27 mm GU-46 for the second suturing). First, the mucosal defect was closed completely in the same manner as for normal endoscopic hand-suturing, using a weakly curved needle that was easy to penetrate the mucosa without regard to suturing length. In the second suture, a strong double-curved needle was used to cross over the first suture line ( Fig. 1 , Fig. 2 , Fig. 3 ).

**Fig. 1:**
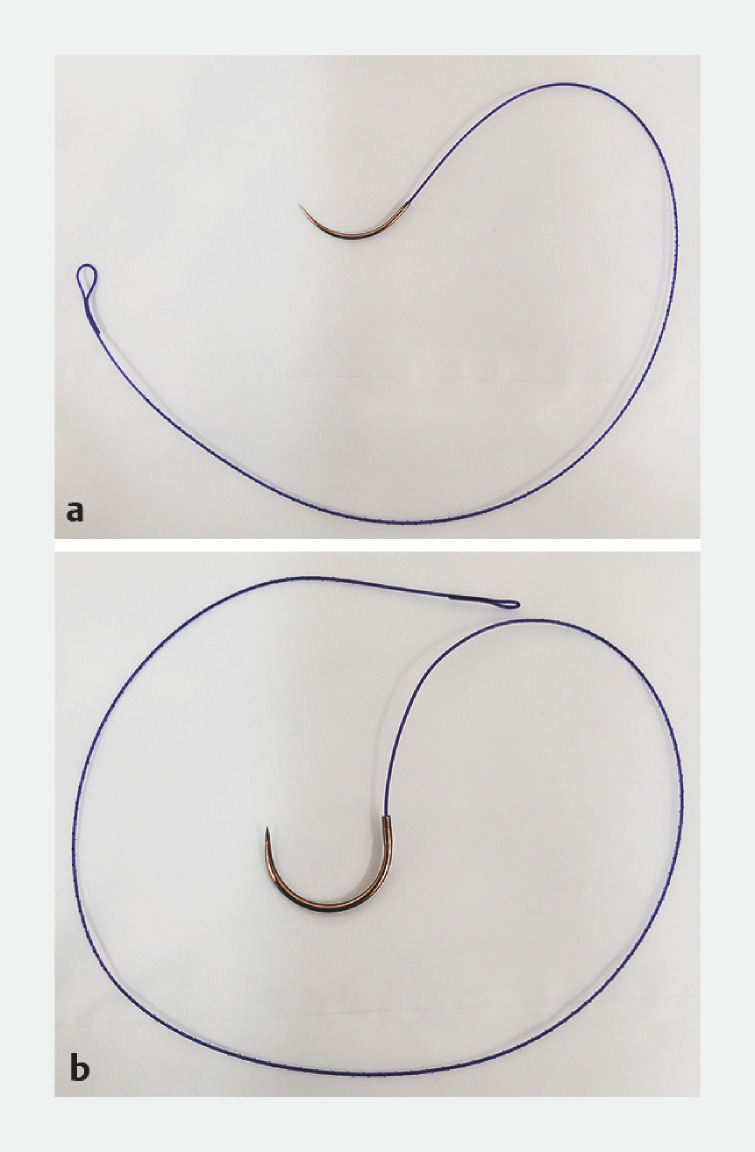
Needles used for double hand suture. **a** A weakly curved needle. **b** A strong double-curved needle.

**Fig. 2:**
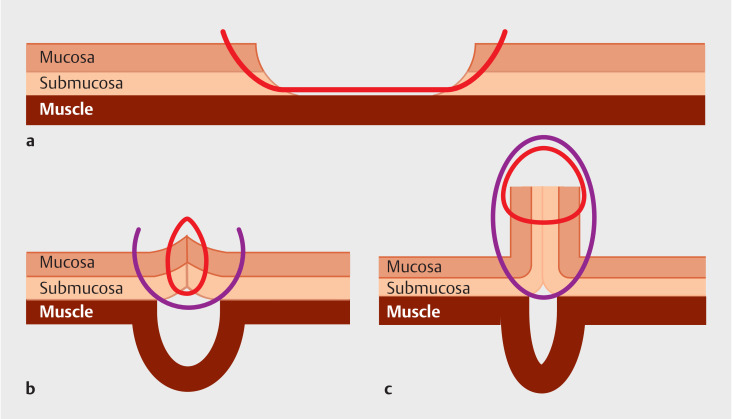
Schematic of the endoscopic hand double suture: red line is the first suture; purple line is the second suture. **a** Complete closure of the mucosal defect in the same manner as for normal endoscopic hand-suturing procedure, using a weakly curved needle. **b** Closure of only the mucosal defect creates a submucosal dead space. In the second suture, a strong double-curved needle is used to cross over the first suture line. **c** This double suture technique decreases the dead space.

**Fig. 3:**
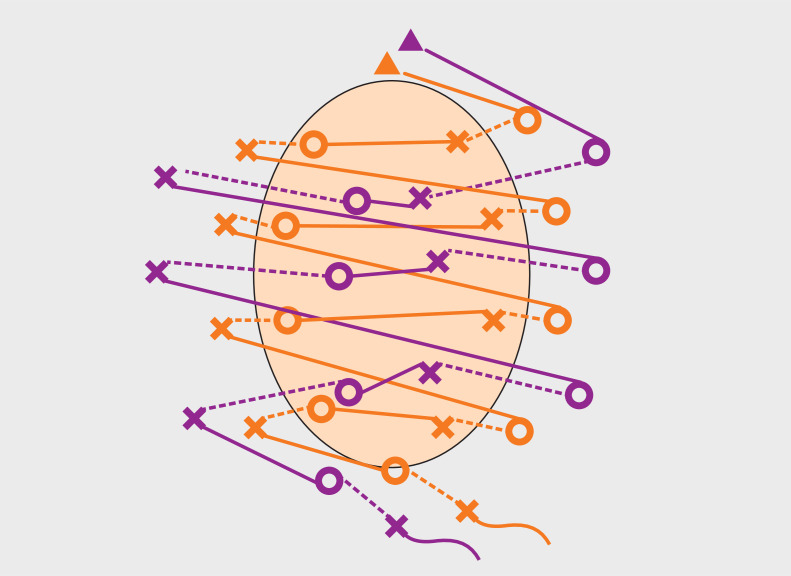
Schematic of the order for endoscopic hand double suture. Triangles are anchors, circles are needle entry points, crosses are needle exit points, dotted lines are threads within the submucosa, and solid lines are threads on the mucosa. Orange is the first suture and purple is the second suture.

An 82-year-old woman was referred to our hospital due to a 10 mm 0-IIa lesion on the anterior wall of the gastric angle. We performed en bloc resection through endoscopic submucosal dissection. Following the excision, double suturing was performed for the mucosal defect ( Fig. 4 **a–d** , Video 1 ). On the first and sixth day after treatment, a follow-up endoscopy was conducted; no wound opening had occurred ( Fig. 5 ) and the patient was discharged.

**Fig. 4:**
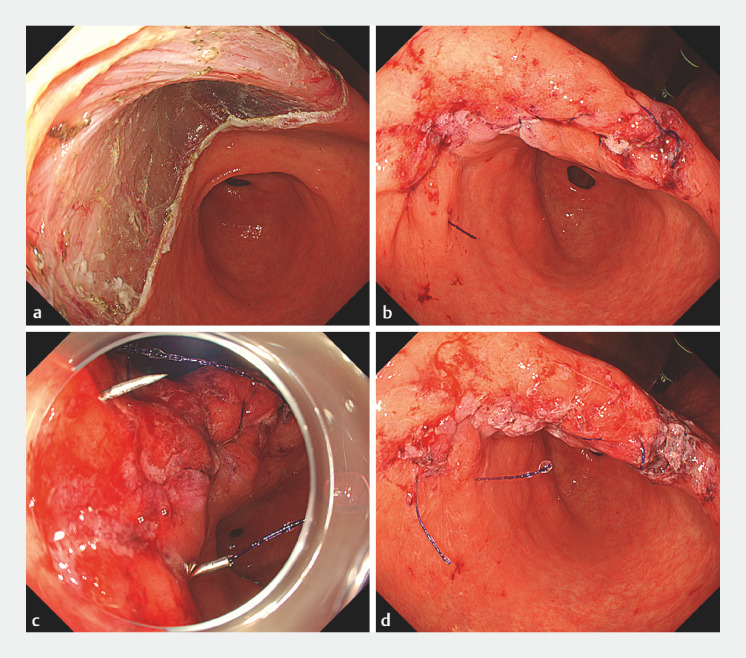
Endoscopic view of double suturing. **a** Mucosal defect after gastric endoscopic submucosal dissection. **b** Achieving complete closure after the first suture. **c** The second suture with a strong double-curved needle. **d** Complete closure was reinforced with double sutures.

**Fig. 5:**
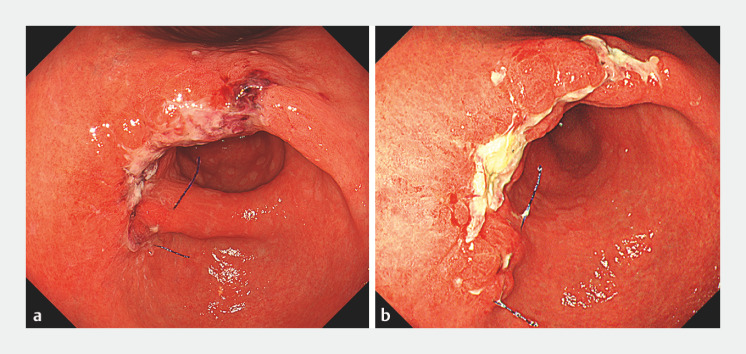
Endoscopic view after the double suturing. **a** The next day. **b** After 6 days.

Innovative endoscopic hand double suturing technique for mucosal closure after gastric endoscopic submucosal dissection.Video 1

Endoscopic double suturing not only reinforces suture strength, but also reduces dead space, making wound dehiscence less likely and potentially reducing the risk of postoperative adverse events.

Endoscopy_UCTN_Code_TTT_1AO_2AD

## Notes

Endoscopy E-Videos https://eref.thieme.de/e-videos
*E-Videos* is an open access online section of the journal *Endoscopy* , reporting on interesting cases and new techniques in gastroenterological endoscopy. All papers include a high-quality video and are published with a Creative Commons CC-BY license. Endoscopy E-Videos qualify for HINARI discounts and waivers and eligibility is automatically checked during the submission process. We grant 100% waivers to articles whose corresponding authors are based in Group A countries and 50% waivers to those who are based in Group B countries as classified by Research4Life (see: https://www.research4life.org/access/eligibility/ ). This section has its own submission website at https://mc.manuscriptcentral.com/e-videos .
